# Mitochondrial Targeting of Herbal Medicine in Chronic Kidney Disease

**DOI:** 10.3389/fphar.2021.632388

**Published:** 2021-05-26

**Authors:** Qing Li, Changying Xing, Yanggang Yuan

**Affiliations:** Department of Nephrology, the First Affiliated Hospital of Nanjing Medical University, Nanjing Medical University, Nanjing, China

**Keywords:** chronic kidney disease, herbal medicine, mitochondria, ROS, mitochondrial dysfunction

## Abstract

Chronic kidney disease (CKD) is a common progressive disease that is typically characterized by the permanent loss of nephrons and an eventual decline in glomerular filtration rate. CKD increases mortality and has a significant impact on the quality of life and the economy, which is becoming a major public health issue worldwide. Since current conventional-medicine treatment options for CKD are not satisfactory, many patients seek complementary and alternative medicine treatments including Traditional Chinese Medicine. Herbal medicine is often used to relieve symptoms of renal diseases in the clinic. The kidney is abundant in the number of mitochondria, which provide enough energy for renal function and metabolism. In recent years, a vital role for mitochondrial dysfunction has been suggested in CKD. Mitochondria have become a new target for the treatment of diseases. A growing number of studies have demonstrated herbal medicine could restore mitochondrial function and alleviate renal injury both *in vivo* and *in vitro*. In this review, we sum up the therapeutic effect of herbal medicine in CKD via targeting mitochondrial function. This implies future strategies in preventing CKD.

## Introduction

Chronic kidney disease (CKD) is a major public health issue worldwide, which is related to severe complications, poor quality of life and increased financial burden (Bello et al., 2017). CKD is defined as a gradual decrease in renal function and eventually resulting in end-stage renal disease (ESRD). The main aims of treatment are to slow the progression of the decline in renal function and limit extrarenal complications (Nasrallah et al., 2014). However, the current therapeutic approaches for CKD have not achieved the desired effects in the clinic (Leporini et al., 2016).

The search for complementary and alternative medicine treatments for CKD is thus arousing more and more attention. In the clinic, many patients seek Traditional Chinese Medicine for alternative therapy. Traditional Chinese Medicine plays an important role in the Chinese healthcare system that has been developed over the centuries to prevent and treat diseases. It includes mind-body practices and herbal medicines (Spatz et al., 2018). Herbal medicine is abundant in various chemical components, such as active substances with therapeutic value and nutritional active ingredients, which determines the characteristics of its multi-target. Studies showed that herbal medicine could relieve symptoms, decrease proteinuria, and slow the reduction of renal function (Zhong et al., 2015).

Mitochondria provide energy for the kidney to clear waste in the blood and regulate water and electrolyte balance (Sun et al., 2019). Mitochondrial dysfunction is closely related to the development and progression of CKD. The maintenance of mitochondrial function relies on the balance of mitochondrial turnover and mitochondrial dynamics (Aparicio-Trejo et al., 2018). Mitochondrial turnover includes mitochondrial biogenesis, which augments mitochondrial mass and ATP production, and mitophagy that removes damaged mitochondria (Pedraza-Chaverri et al., 2016). Mitochondria are in a dynamic equilibrium of fission and fusion to maintain mitochondrial homeostasis.

**FIGURE 1:**
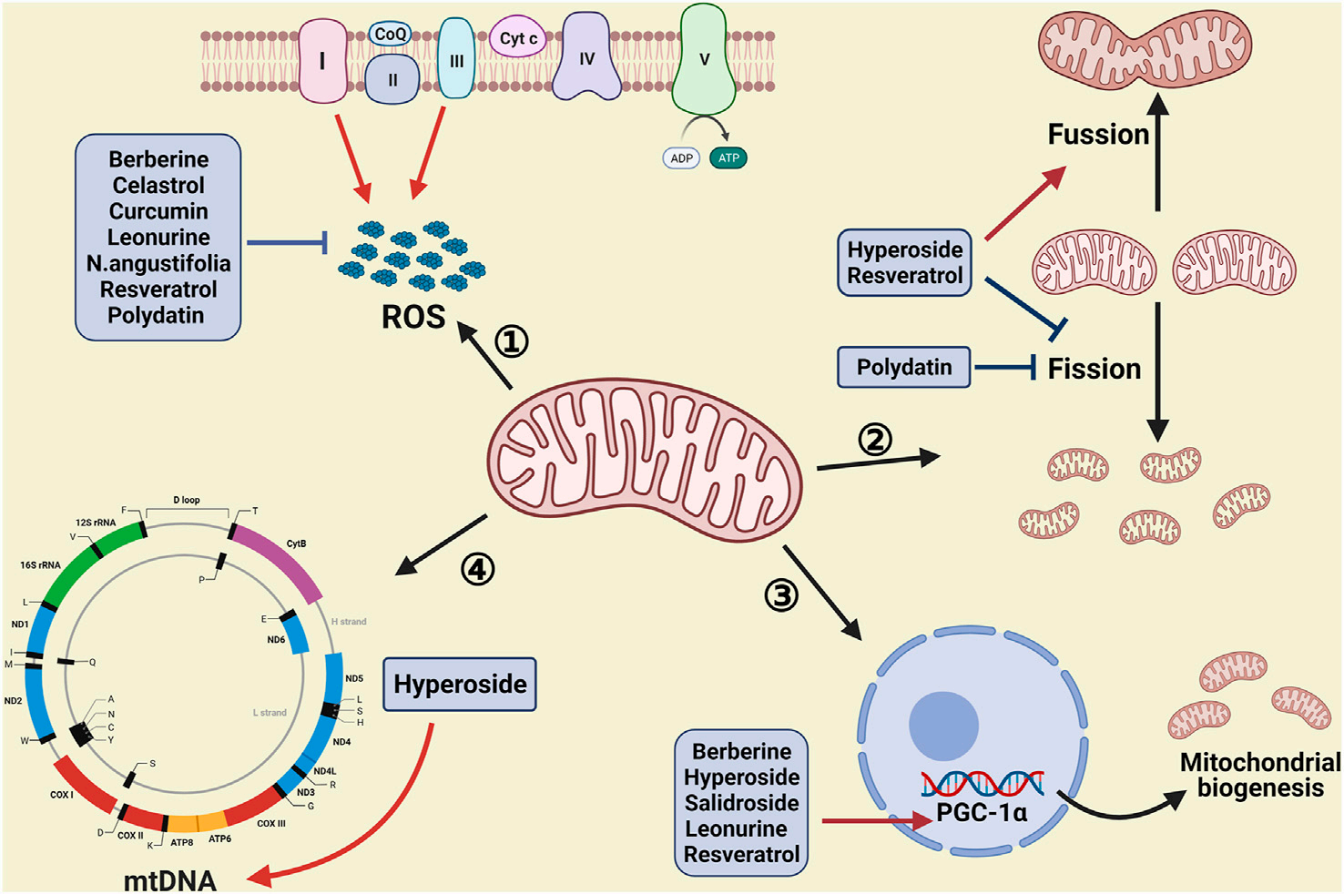
Therapeutic interventions of herbal medicine by targeting mitochondrial homeostasis. Many procedures are involved in mitochondrial homeostasis, such as the production of ROS, mitochondrial dynamics and mitochondrial biogenesis. Herbal medicine plays a protective role in CKD via decreasing the augmentation of ROS, inhibiting mitochondrial fission, promoting mitochondrial fusion, as well as increasing mitochondrial biogenesis and the copies of mtDNA (Created with BioRender.com).

Although a vast amount of evidence showed herbal medicines could alleviate renal injury by regulating mitochondrial function, a more detailed understanding of the role of mitochondrial homeostasis in herbal medicines is still limited. This review pays attention to the therapeutical effect of herbal medicines in CKD by targeting mitochondrial function, especially the application prospect of herbal medicines (Table 1 and Figure 1).

**TABLE 1:** Mitochondria-targeted herbs in chronic kidney disease.

Targets	Herbs	Detection indicator	*In vivo*	*In vitro*	References
Reactive oxygen species	Berberine	↓ROS,↑SOD,↑GSH	—	In HG-treated NRK-52E and HK2 cells	Zhang et al. (2016)
		↓ROS	In db/db mice	In PA-treated mouse podocytes	Qin et al. (2020)
	Betulinic acid	↑SOD	In diabetic rats	—	Xie et al. (2017)
	Celastrol	↓ROS	—	In HG-treated MPC5 cells	Zhan et al. (2018)
	Curcumin	↓ROS,↑SOD	In STZ-treated rats	In HG-treated MPC5 cells	Sun et al. (2016)
	Leonurine	↓ROS	In ADR-treated mice	In ADR-treated podocytes	Liu et al. (2018)
	N. angustifolia	↓ROS ,↑SOD,↑GSH	In diabetic rats	In H_2_O_2_-treated HBZY-1 cells	Huang et al. (2020)
	Polydatin	↓ROS	—	In HG treated MPC5 cells	Zheng et al. (2017)
		↓ROS,↑SOD1	In STZ-treated mice	In HG treated GMCs	Gong et al., 2017)
	Resveratrol	↓ROS	In 5/6 nephrectomized rats	In TGF-β1-treated mouse mesangial cells	Hui et al. (2017)
		↑SOD1,↑SOD2	In aged mice	In H_2_O_2_-treated HK2 cells	Kim et al. (2018)
Mitochondrial biogenesis	Berberine	↑PGC1-α	In db/db mice	In PA-treated mouse podocytes	Qin et al. (2020)
	Hyperoside	↑PGC1-α	In ADR-treated mice	In ADR-treated podocytes	Chen et al. (2017)
	Leonurine	↑PGC1-α	In ADR-treated mice	In ADR-treated podocytes	Liu et al. (2018)
	Resveratrol	↑PGC1-α	In aged mice	—	Kim et al. (2018)
	Salidroside	↑PGC1-α	In STZ-treated mice	—	Xue et al. (2019)
Mitochondrial fusion	Hyperoside	↑Mfn1	In ADR-treated mice	In ADR-treated podocytes	Chen et al. (2017)
	Resveratrol	↑Mfn2,↑OPA1	In 5/6 nephrectomized rats	In TGF-β1-treated mouse mesangial cells	Weikang et al. (2017)
Mitochondrial fission	Hyperoside	↓Drp1,	In ADR-treated mice	In ADR-treated podocytes	Chen et al. (2017)
	Polydatin	↓Drp1,↓p-Drp1	In KKAy mice	In HG-treated MPC5 cells	Lin et al. (2017)
	Resveratrol	↓Drp1	In 5/6 nephrectomized rats	In TGF-β1-treated mouse mesangial cells	Hui et al. (2017)
Mitochondrial DNA	Hyperoside	↑mtDNA	In ADR-treated mice	In ADR-treated podocytes	Chen et al. (2017)

### Herbal Medicines in CKD via Targeting Mitochondrial Biogenesis

Mitochondrial biogenesis is a complicated process that new mitochondria are generated from preexisting mitochondria to increase mitochondrial mass. Mitochondrial biogenesis mainly includes four parts: the inner and outer mitochondrial membranes synthesis, synthesis of the proteins encoded by mitochondria, synthesis and imported mitochondrial proteins encoded by nuclear, as well as replication of mitochondrial DNA (mtDNA) (Li et al., 2017; Fontecha-Barriuso et al., 2020). Peroxisome proliferator-activated receptor γ coactivator-1α (PGC-1α) is the key regulator of mitochondrial biogenesis which can not only improve the expression of nuclear respiratory factor 1 and 2 (NRF1 and NRF2) but also enhance the interaction with them. The activation of NRF1 and NRF2 stimulates mtDNA replication and transcription via mitochondrial transcription factor A (Tfam). Also, the activation of NRF1 and NRF2 increases the transcription of subunits of mitochondrial electron transport chain encoded by nuclear (Uittenbogaard and Chiaramello, 2014; Li et al., 2017). Berberine, a naturally occurring isoquinoline alkaloid, is present in Coptidis rhizoma and Cortex Phellodendri (Zhang et al., 2016). Qin et al. reported that PGC-1α was significantly decreased in db/db mice and palmitic acid-induced podocytes. Berberine increased the expression of PGC-1α and improved mitochondrial function via the AMPK/PGC-1α pathway both *in vivo* and *in vitro* (Qin et al., 2020). Hyperoside, also named quercetin 3-o- β -D-galactopyranoside, is a flavonol glycoside extracted from *Rhododendron ponticum* L that is widely used in Traditional Chinese Medcine (Li et al., 2013; Wang et al., 2018). Chen et al. proposed hyperoside improved the loss of PGC-1α and mitochondrial dysfunction caused by adriamycin both *in vivo* and *in vitro* (Chen et al., 2017). Leonurine (C14H21N3O5), a natural alkaloid, is derived from Herba Leonuri (Hu et al., 2019; Zhang et al., 2019). Leonurine could ameliorate the expression of PGC-1α in adriamycin-treated podocytes (Liu et al., 2018). Resveratrol is a polyphenolic phytoalexin belonging to the stilbenes derivative which is abundant in berries, grapes, nuts (Asgary et al., 2019). Kim et al. observed that resveratrol increased the protein level of PGC-1α and Nrf2 and ameliorated mitochondrial dysfunction in aging-related renal injury (Kim et al., 2018). Salidroside is a bioactive compound isolated from Traditional Chinese Medicine Rhodiola rosea (Zhao et al., 2019). Xue et al. proposed salidroside attenuated the damage of renal structure and function in STZ-induced diabetic mice. Furthermore, the decrease of PGC-1α was reversed in salidroside-treated mice (Xue et al., 2019).

### Herbal Medicines in CKD via Targeting Mitochondrial Dynamics

Mitochondria are continuously changing their morphology by constant fission and fusion to adapt to environmental imperatives and cellular energetic needs (Rovira-Llopis et al., 2017). The disruption between the balance of fission and fusion leads to mitochondrial dysfunction and eventually evokes the development of mitochondria-related diseases. Mitochondrial dynamics depend on several dynamin-related guanosine triphosphatases (GTPases). Mitochondrial fission, split one mitochondrion into two daughter mitochondria, is mainly regulated by Dynamin-related protein 1 (Drp1) in the cytosol and its receptors on the outer membrane, including fission protein 1 (Fis1), mitochondrial fission factor (MFF) and mitochondrial dynamics proteins of 49 kDa and 51 kDa (MiD49 and MiD51) (Benigni et al., 2016). Mitochondrial fusion, combine two mitochondria into one mitochondrion, is a two-step process resulting from its double membranes. Mitofusin1 and 2 (Mfn1/2) contribute to outer-membrane fusion, while Optic Atrophy 1 (OPA1) mediates the fusion of the inner membrane (Mishra and Chan, 2016). Chen et al. showed adriamycin triggered mitochondrial fission and reduced the expression of Mfn1, which were reversed after treating with hyperoside (Chen et al., 2017). Polydatin is a monocrystalline and polyphenolic drug extracted from the traditional herb Polygonum cuspidatum (Gao et al., 2015). Zheng et al. pointed that polydatin ameliorated renal function in KKAy mice and reduced apoptosis in KKAy mice and high glucose-exposed podocytes. In addition, they found polydatin decreased the elevation of p-Drp1 and fragmented mitochondria caused by high glucose. Furthermore, they demonstrated the upregulation of Drp1 by transfection with Drp1-GFP lentivirus enhanced apoptosis and fragmented mitochondria while polydatin blocked the increase of apoptosis (Zheng et al., 2017). Hui et al. showed the expressions of Mfn2 and OPA1 were declined but the level of Drp1 was increased in 5/6 nephrectomized rats model and TGF-β1-treated mesangial cells. Resveratrol treatment reversed these changes and restored mitochondrial function both *in vivo* and *in vitro* (Hui et al., 2017).

### Herbal Medicines in CKD via Targeting Mitochondrial ROS

Reactive oxygen species (ROS), byproducts of cellular aerobic metabolism, play a significant part in cellular signaling processes. ROS consists of superoxide anion (O_2_^•−^), hydroxyl radicals (OH^•^), and hydrogen peroxide (H_2_O_2_) (Schieber and Chandel, 2014). As the main production of endogenous ROS, mitochondria consumed 1–2% of oxygen to produce ROS in the process of mitochondrial respiration (Pinegin et al., 2018). It has been reported that complex I and complex III of the mitochondrial electron transport chain are the major sites of the production of ROS in mitochondria (Mazat et al., 2020). ROS produced by complex I (NADH: ubiquinone oxidoreductase) is mainly in the mitochondrial matrix, while ROS created by complex III (ubiquinol: cytochrome oxidoreductase) is both in the matrix and intermembrane space. Mitochondrial matrix dehydrogenases contribute a lot to the generation of ROS as well (Pinegin et al., 2018; Stefanatos and Sanz, 2018; Bouchez and Devin, 2019). Scavenging of ROS contributes to the homeostasis of ROS in mitochondria. The O_2_^•−^ produced in the mitochondrial matrix is dismutated to H_2_O_2_ by superoxide dismutase protein 2 (SOD2). The O_2_^•−^ generated in intermembrane space is converted to H_2_O_2_ by SOD1 (Diebold and Chandel, 2016). H_2_O_2_ is degraded to H_2_O by glutathione peroxidases (GPX) or peroxiredoxins (PRX). The excessive production of mitochondrial ROS or the impaired antioxidant defense leads to mitochondrial dysfunction and renal injury (Angelova and Abramov, 2018). Zhang et al. discovered, in high glucose treated cultured tubular epithelial cell line and human kidney proximal tubular cell line, berberine mitigated ROS production and elevated the level of GSH and SOD by PI3K/Akt pathway (Zhang et al., 2016). In palmitic acid-induced podocytes, berberine attenuated the increased generation of ROS reflected by MitoSOX Red (Qin et al., 2020). As a plant-derived pentacyclic lupane-type triterpenoid, betulinic acid can be extracted from white birch trees (Phillips et al., 2018; Ríos and Máñez, 2018). Xie et al. proposed betulinic acid raised the expression of SOD through AMPK/NF-κB/Nrf2 pathway in streptozotocin-induced diabetic rats (Xie et al., 2017). Celastrol, a naturally occurring pentacyclic triterpene, is extracted from Tripterygium wilfordii (Fang et al., 2019). Zhan et al. found treatment with celastrol decreased the production of ROS in high glucose-treated podocytes (Zhan et al., 2018). Curcumin, a natural polyphenol, is an active component of turmeric that has been used as a spice and traditional medicine for centuries in India and China (Bahrami et al., 2019). Sun et al. discovered the level of ROS was escalated in a time-dependent way in high glucose-induced podocytes. In contrast, the activity of SOD was gradually reduced in podocytes exposed to high glucose in a time-dependent manner. Curcumin inhibited ROS and improved the activity of SOD in diabetic nephropathy both *in vivo* and *in vitro* (Sun et al., 2016). Liu et al. found leonurine alleviated the augmentation of ROS and increased the level of SOD2 in adriamycin-induced podocyte injury (Liu et al., 2018). Nepeta angustifolia C. Y. Wu, a traditional Tibetan medicine, consists of pentacyclic triterpenes and bioactive flavonoids which are reported to have anti-inflammatory and antioxidant effects (Huang et al., 2019). In the kidney of diabetic rats, the expressions of SOD and GSH were remarkably augmented after exposure to Nepeta angustifolia C. Y. Wu. In addition, Nepeta angustifolia C. Y. Wu attenuated ROS production in H_2_O_2_-induced rat glomerular mesangial cells. Compared with the group treated with H_2_O_2_, Nepeta angustifolia C. Y. Wu also improved the expression of SOD in rat glomerular mesangial cells (Huang et al., 2020). Polydatin, a resveratrol glycoside, ameliorated ROS levels in high glucose-treated primary podocytes and glomerular mesangial cells. Additionally, Gong et al. pointed out that polydatin increased the activity of SOD in the kidney and serum of diabetic mice (Gong et al., 2017; Zheng et al., 2017). Hui et al. observed resveratrol reversed the enhancement of ROS in 5/6 nephrectomized rats and TGF-β1-treated mouse mesangial cells (Hui et al., 2017). Furthermore, resveratrol improved SOD1 and SOD2 in aged mice and H_2_O_2_-treated human kidney proximal tubular cells by activating Nrf2 and SIRT1 pathways (Kim et al., 2018).

### Herbal Medicines in CKD via Targeting Mitochondrial DNA

Mitochondrial DNA (mtDNA), consisting of 16,569 base pairs in humans, is a circular double-stranded genome (El-Hattab et al., 2017). mtDNA carries 37 genes containing 13 polypeptides, 22 tRNAs and 2 rRNAs (Srirattana and St. John, 2019). The proteins encoded by 13 polypeptides are major parts of mitochondrial oxidative phosphorylation including 7 subunits of Complex I, 1 of Complex III, 3 of Complex IV, and 2 of Complex V (Roubicek and Souza-Pinto, 2017). Apart from them, other proteins mitochondria needed are encoded by nuclear, synthesized in the cytoplasm, and eventually transported into mitochondria (Szczepanowska and Trifunovic, 2015). The mtDNA is coated with proteins and organized in a complex named mitochondrial nucleoid. Mitochondrial nucleoids are located on the matrix side of the mitochondrial inner membrane and well-distributed in the mitochondria for cellular metabolic needs (Almannai et al., 2018). The copy number of mtDNA is the indicator of mitochondrial function (Tin et al., 2016). In adriamycin (Chen et al., 2017) mice, the level of urinary albumin (Chen et al., 2017) was lifted while mtDNA was significantly reduced. Compared with the adriamycin group, the group exposed to hyperoside exhibited lower urinary albumin and increased mtDNA. In addition, co-incubated with hyperoside augmented the copies of mtDNA in adriamycin-induced human podocytes (Chen et al., 2017).

There are some limitations in this review that merit attention. First, herbal medicine usually has multiple targets. Further studies are needed to confirm whether mitochondria are the specific target. Second, very little is known about how herbal medicine enters the mitochondria and regulates mitochondrial homeostasis. Third, most of the Traditional Chinese Medicine we mentioned above showed protective effects in animal and cellular experiments, it remains unclear if they could be applied to clinical trials.

## Conclusion

Recently, more and more attention has been paid to the role of mitochondria in the occurrence and development of chronic kidney disease. A growing number of studies have proved mitochondria gradually became the promising target for the therapy of CKD. Traditional Chinese Medicine targeting mitochondria provides new ideas for the clinical treatment of CKD.
